# Feasibility of Pastel Nagomi Art for Older Adults with Psychiatric Disorders and Dementia

**DOI:** 10.1192/j.eurpsy.2026.11570

**Published:** 2026-07-31

**Authors:** W. Peng

**Affiliations:** Taipei City Hospital, Taipei, Taiwan

## Abstract

**Introduction:**

Art therapy has been shown to enhance emotional expression and self-expression in patients with schizophrenia and mood disorders, improving symptom management. In addition, art therapy for older adults with dementia can increase engagement and feasibility in activities. Pastel Nagomi Art, a Japanese pastel technique applied with fingers, requires no prior artistic experience or special tools. It is easy to learn, promotes relaxation and attention span, and results in attractive artwork that fosters a sense of accomplishment.

**Objectives:**

This study explored the feasibility of Pastel Nagomi Art sessions for older adults with psychiatric disorders and dementia.

**Methods:**

Sixteen Pastel Nagomi Art sessions were conducted with 15 older adults in a hospital day-care ward, including participants diagnosed with schizophrenia, bipolar disorder, depression, anxiety, or dementia. Feasibility was assessed by observing engagement, duration of attention, artwork completion, and creativity.

**Results:**

Across the 16 sessions, attendance averaged 80%, with all attendees completing their artwork. Participants sustained attention for over 30 minutes during activities. In later sessions, participants showed increased initiative in creating artwork and greater autonomy in color selection and content.

**Conclusions:**

Pastel Nagomi Art sessions appear feasible and suitable for older adults with psychiatric disorders and dementia, supporting their potential as a therapeutic art intervention.

**Disclosure of Interest:**

None Declared

